# The effects of genre on the lexical richness of argumentative and expository writing by Chinese EFL learners

**DOI:** 10.3389/fpsyg.2022.1082228

**Published:** 2023-01-10

**Authors:** Renquan Heng, Liping Pu, Xing Liu

**Affiliations:** ^1^School of Foreign Languages, Soochow University, Suzhou, China; ^2^Soochow College, Soochow University, Suzhou, China

**Keywords:** richness, argumentative composition, expository composition, Chinese EFL students, genre

## Abstract

Lexical richness, a crucial aspect of L2 writing research, has been shown to make a difference in L2 writing performance. Nonetheless, the majority of empirical studies have focused either on a single text type or on the comparison between narrative and non-narrative writing (mostly argumentative writing) in academic contexts, whereas there has been a dearth of research regarding the lexical features pertaining to varied non-narrative writing genres. Considering the cognitive demands intrinsic in different writing task types, this study examined the development of lexical richness, which includes lexical density, lexical variation, and lexical sophistication, in Chinese EFL students’ argumentative and expository compositions over the course of one academic year. Fifty-four participants were asked to write eight compositions (in two alternating genres)—four argumentative and four expository—which were parsed using two computational tools. The results indicated a significant increase in all three subconstructs of lexical richness in argumentative compositions over the year, while in expository compositions, only lexical density and lexical sophistication demonstrated an increasing trend. As time went on, the participants in both genres tended to use more high-frequency words with more senses, more academic words, more high-frequency bigrams, and words that are less familiar and more precise. Moreover, the argumentative compositions displayed higher lexical density than the expository ones, while the expository compositions manifested greater lexical variation and lexical sophistication than the argumentative ones. The findings of the study suggest some implications for L2 writing teaching and research.

## 1. Introduction

Vocabulary is considered to be at the heart of meaning-making in understanding discourse (Halliday and Hasan, 1976), and having a rich and complex vocabulary is viewed as a crucial component that contributes to the quality of writing for academic purposes (Maamuujav, 2021). In the past few decades, research has shown that the richness of lexis, or rather lexical richness, makes an important contribution to second language (L2) writing quality (Jarvis et al., 2003; Olinghouse and Leaird, 2009; Ha, 2019), and the ability to produce a well-written text is thought to be important to individual success both at school and in the workplace (Powell, 2009). However, it is arduous and challenging for both L1 (first language) and L2 learners to become an advanced writer, because students should, apart from having a good command of vocabulary, learn to compose different genres of writing which require them to employ varied skills and linguistic resources (Pu et al., 2022). In recent years, there has been a growing increase in research that explores language development across writing genres (Ravid, 2004; Beers and Nagy, 2011; Qin and Uccelli, 2016; Jeong, 2017; Yoon and Polio, 2017; Bi, 2020), and it has been found that in both L1 and L2 writing, argumentative compositions tend to be more linguistically complex than narrative compositions. However, previous studies mostly focused on a single text type or on comparisons between narrative and non-narrative writing (mostly argumentative writing) in academic contexts without examining the lexical features pertaining to varied non-narrative writing genres (Pu et al., 2022).

Research has demonstrated that L2 writers deploy different linguistic features for different levels of cognitive demands intrinsic to narrative, argumentative, and expository tasks (Weigle, 2002; Kormos, 2011). In relation to this, there are two conflicting hypotheses: Robinson’s (2001) Cognition Hypothesis and Skehan’s (1998) Limited Attentional Capacity Model. The former claims that a more complex task will lead to more complex language and greater accuracy, whereas the latter asserts that a speaker’s cognitive capacity is limited, so a more complex task will result in less complex and less accurate language. Both L1 and L2 studies have already yielded higher lexical richness in non-narrative writing than in narrative writing (Ravid, 2004; Qin and Uccelli, 2016; Yoon and Polio, 2017). Moreover, while previous studies have mostly relied on surface measures and investigated broad linguistic features to describe, distinguish and explain the degree of proficiency exhibited in texts written by non-native speakers of English (Halliday and Hasan, 1976; Wolfe-Quintero et al., 1998; Crossley and Skalicky, 2019), this study, by way of employing finer-grained indices to gauge lexical richness, will provide a new perspective for both teachers and students to know about the development of lexical richness in L2 learners’ writing. To venture in this direction, the present study intends to determine whether lexical richness develops uniformly or differently in argumentative and expository writing over one academic year and whether there are genre effects on the lexical features of different writing task types.

## 2. Literature review

### 2.1. Lexical richness

Lexical richness is a crucial aspect of L2 writing research, and a sophisticated, diverse, and accurate lexical contribution to texts enhances writing quality and showcases the learner’s writing proficiency (Zhang and Daller, 2020; Zhang et al., 2021). Nonetheless, there has been no consensus in the literature regarding the conceptualisation of lexical richness (Laufer and Nation, 1995; Jarvis, 2013; Treffers-Daller et al., 2018; Zhang et al., 2021). Some studies equate lexical richness with a variety of different words (Kalantari and Gholami, 2017), while for others, it is a multidimensional concept (Laufer and Nation, 1995; Wolfe-Quintero et al., 1998; Read, 2000; Lu, 2012). For instance, Daller et al. (2003) equated lexical richness to lexical diversity and lexical complexity, while Laufer and Nation (1995) examined it in terms of lexical variation, lexical density, lexical sophistication, and lexical originality. However, Read (2000) contended that lexical originality could not evaluate the development of lexical performance; therefore, he proposed modifying Laufer and Nation’s categorization by taking error into consideration, rendering lexical richness in the form of lexical sophistication, lexical variation, lexical density, and lexical errors. Also taking errors into consideration, Engber (1995) suggested that lexical richness includes lexical variation with errors, lexical variation without errors, percentage of lexical errors, and lexical density. Although they differ in their views on the subcomponents of lexical richness, most researchers have, by and large, focused their attention on three main components: lexical density, lexical variation, and lexical sophistication (Laufer and Nation, 1995; Qin and Wen, 2007; Lu, 2012; Bulté and Housen, 2014). The present study follows the multidimensional model and explores lexical features along the three most often researched dimensions, namely lexical density, lexical variation, and lexical sophistication, in argumentative and expository compositions written by Chinese EFL learners over the course of one academic year.

### 2.2. Argumentative writing and expository writing

Text genres are primarily divided into narratives and non-narratives in both academic and non-academic contexts (Bruner, 1986; Pu et al., 2022). Narratives focus on events and actions in settings performed by the characters, whereas non-narratives (e.g., argumentative, expository, and descriptive) focus on ideas and concepts and express the unfolding of claims and argumentation in a logical fashion (Berman and Slobin, 1994; Tian, 2014). Among non-narratives, argumentation mainly invites a writer to give personal opinions and judgment on a debatable issue or statement and to take a stand on the issue or statement based on facts, generalizations, and reasoning, while exposition mainly invites a writer to explain and provide information about something (not to take a side on something debatable or to argue on the topic), based on facts and generalizations of events and states (Genung, 1900; Yang, 2014).

Genre is concerned with cognitive task complexity, which, in turn, is related to two competing hypotheses—Cognition Hypothesis (Robinson, 2001, 2003) and the Limited Attentional Capacity Model (Skehan, 1998). In general, different genres place different levels of cognitive demands on learners, with narrative being the least cognitively demanding, exposition being more cognitively demanding than narrative, and argumentation being the most cognitively complex (Weigle, 2002). In light of varied cognitive demands, different genres may exhibit distinct language characteristics as a way to describe and clarify ideas and expressions in different types of writing (Ravid, 2004; Pu et al., 2022). This is the basic motive for the present study, i.e., to compare and contrast the developmental features of lexical richness in argumentative and expository compositions by Chinese EFL learners over the course of one academic year.

### 2.3. Studies on lexical richness in relation to genre differentiation

Research has shown that writing across genres involves different cognitive task loads and requires different linguistic demands (Berman and Nir-Sagiv, 2007; Beers and Nagy, 2011; Kormos, 2011; Pu et al., 2022). Moreover, differences among genres are not only restricted to macro-structural elements (Beers and Nagy, 2009; Biber and Conrad, 2009; Beers and Nagy, 2011; Lu, 2011), but they can also occur at the word level (Bar-Ilan and Berman, 2007). L1 studies on children’s writing have documented that argumentative or expository compositions have higher lexical richness than narrative ones. Ravid (2004) examined the differences across Hebrew L1 narrative and expository compositions produced by child, adolescent, and adult writers of Hebrew. The results demonstrate a greater lexical density for expository compositions than for narrative ones. Similarly, Berman and his colleagues (Bar-Ilan and Berman, 2007; Berman and Nir-Sagiv, 2007) conducted studies across seven languages comparing writing in two genres (narrative and expository) by children at three different ages, testifying to consistent differences between narrative and expository compositions. Likewise, Olinghouse and Wilson (2013) examined the features of lexis in story, persuasive, and informative texts written by 105 English fifth graders. They found that story texts had higher diversity and maturity than informative or persuasive texts.

As for studies on lexical richness in L2 writing, they are mostly focused on one single text type or on the comparison between narrative and non-narrative writing (mostly argumentative writing) in academic contexts (Lu, 2011; Qin and Uccelli, 2016; Yıldız and Yeşilyurt, 2017; Yoon and Polio, 2017; Ha, 2019; Bi, 2020; Lei and Yang, 2020; Azadnia, 2021). Among the studies that focused on a single text type, Ha (2019) researched the contribution of lexical richness to L2 writing quality in argumentative compositions written by thirty-five Korean undergraduates in the final exam of a reading and writing class. By way of the correlation analysis, the study concluded a close relationship between writing quality and the indices of lexical diversity, sophistication, and fluency; in particular, lexical sophistication was found to be the most significant predictor contributing to writing quality. Similarly, Azadnia (2021) examined the lexical richness of a corpus composed of doctoral dissertations written by Iranian TEFL students in terms of lexical density, diversity, and sophistication. The corpus in the study was analyzed in comparison to an L1 baseline containing doctoral dissertations written by native English speakers. Their findings revealed that the texts written by Iranian TEFL learners were lexically less diverse but more sophisticated.

Among studies focusing on the comparison between different genres, Yıldız and Yeşilyurt (2017) carried out a study of 41 Turkish students who were learning English to assess the effects of task planning and rhetorical mode (e.g., descriptive and narrative compositions) on lexical and syntactic complexity, as well as overall writing quality. The results reveal that lexical density, lexical variation, and lexical sophistication in descriptive (or expository) writing were significantly greater than those in narrative writing. Similarly, Qin and Uccelli (2016) compared lexico-syntactic, and genre-specific discourse features in argumentative and narrative compositions written by secondary school Chinese EFL students. Their results show that argumentative compositions displayed higher lexical diversity and lexical sophistication than narrative ones. Likewise, Yoon and Polio (2017) examined lexical richness in the narrative and argumentative genres of writing by ESL learners over one semester. The findings indicate that genre was strongly related to lexical richness, as the writers used longer and less frequent words in argumentative writing and a greater diversity of words in narrative writing. Moreover, Bi (2020) examined genre differences among three proficiency groups and found that for beginner learners, their argumentative writing showed higher lexical richness than narrative writing, but for intermediate and advanced learners, they were apt to use more sophisticated and more diverse words in narrative writing.

A review of previous studies on lexical richness in both L1 and L2 literature shows that few studies have been conducted to explore the differences across non-narrative genres of writing. Moreover, most related studies are cross-sectional, where lexical richness is examined across different grades or proficiency levels at a given point in time (Bi, 2020; Zhang et al., 2021). In view of this, the present study intends to address the following two research questions: (1) What are the changes in lexical richness over the course of one academic year in Chinese EFL learners’ argumentative and expository compositions? (2) What are the effects exerted by genre on lexical richness in Chinese EFL learners’ argumentative and expository compositions?

## 3. Methodology

### 3.1. Research context and participants

The study was positioned in a one-year Comprehensive English course at a leading university in Jiangsu Province, mainland China. The course lasted for an entire academic year and the first author of this paper was actually the teacher of this course. In the university where the study was conducted, the participants were supposed to take the TEM-4 (Test for English Majors) at the end of their second-year college study, which is composed of listening, speaking, reading, and writing. The genres of writing for the TEM-4 are mostly argumentative and sometimes expository. To prepare for the test, the participants had a lot of writing practice over the one academic year, which constituted the materials for the study, with the informed consent from all participants. The participants, aged between 19 and 21, came from two parallel classes (with 28 and 31 students respectively) of the same grade. They all had about 10 years of formal English learning experience and were fairly proficient in writing, though with individual differences. All the courses for the two classes, including both compulsory and optional ones, were given by the same teachers, thus avoiding some potential intervening variables. Nonetheless, in the course of data collection, some participants failed to complete all eight assigned compositions, so only 54 (out of 59) participants’ compositions were valid for analysis in the present study.

### 3.2. Writing tasks

Each of the 54 participants in this study produced eight 300-word compositions (four argumentative and four expository) over the course of one academic year, and the compositions were all finished in class and with no access to electronic devices. The writing topics were determined in relation to the contents of the textbooks (Integrated English III and Integrated English IV) and some contemporary issues of the time. In class, after each unit, the students were asked to give presentations on what they had learned. Meanwhile, the students were encouraged to discuss contemporary issues using the words and expressions they had learned from the texts. In this way, the task difficulties were well controlled. The compositions were collected at regular time intervals, with the first, third, fifth, and seventh being argumentative, and the second, fourth, sixth, and eighth expository (see Table 1).

**Table 1:** Writing prompts for argumentative and expository compositions.

Argumentative 1	Some people believe that the best way to learn about life is by listening to the advice of family and friends. Other people believe that the best way to learn about life is through personal experience. Which do you think is preferable? Use specific examples to support your preference.
Expository 1	Many students have to live with roommates while going to school or university. What are some of the important qualities of a good roommate? Use specific reasons and examples to explain why these qualities are important.
Argumentative 2	Some people learn best when a classroom lesson is presented in an entertaining, enjoyable manner. Other people learn best when a lesson is taught in a serious, formal way. Which of these two ways of learning do you prefer? Use specific reasons and details to support your choice.
Expository 2	Every generation of people is unique in important ways. How is your generation different from your parents’ generation? Use specific reasons and examples to explain your answer.
Argumentative 3	A growing number of people, especially the young, like eating at stands or restaurants, while many others prefer to prepare and eat food at home rather than eat out. Discuss both views and give your preference. Use specific reasons and examples to support your answer.
Expository 3	Imagine that you are preparing for a trip. You plan to be away from your home for a year. You need to make some preparations for the trip. What will you take? Explain why this thing/these things is/are important. Use specific reasons and details to support your choice(s).
Argumentative 4	For many university students there are two alternatives:one way is to find a job, and the other is to pursue further study. Both have advantages and disadvantages, and it is difficult to say which is better. Discuss both views and then give your own opinion. Use specific reasons and examples to support your answer.
Expository 4	People attend college or university for many different reasons (for example, new experiences, career preparation, and increased knowledge). Why do you think people attend college or university? Give reasons for your answer(s) and include any relevant examples from your own experience or knowledge.

### 3.3. Computational tools and measures of lexical richness

Kyle, Crossley and their colleagues (Kyle and Crossley, 2015; Kim et al., 2018; Kyle et al., 2018) have conducted a series of studies to validate TAALES (the Automatic Analysis of Lexical Sophistication) and TAALED (the Tool for the Automatic Analysis of Lexical Diversity). They suggested that some indices are stable and reliable and could be generalized to other populations. In light of previous studies and a pilot study, 15 indices were chosen to measure lexical richness in this study, representing different aspects of the multidimensional construct. Table 2 displays detailed information on the 15 indices measured in the present study.

**Table 2:** Indices of lexical richness examined in the study.

Construct	Label	Definition
Lexical density	Nlex/N	The percent of lexical words
Lexical variation	MATTR	Moving average TTR
	Maas	A log correcting measure
	HD-D	The hypergeometric distribution D
	MTLD	The measure of textual lexical variation
Lexical sophistication		
*Frequency*	AWL	Academic word list all
	Bigram	BNC Written bigram frequency
	Written-CW	BNC written frequency CW logarithm
	Trigram	BNC written trigram frequency
*Range*	KFR_CW	Kucera-Francis register range CW
	WR-AW	BNC written range AW
*Word property*	Familiarity	MRC familiarity CW
	Meaningfulness	MRC meaningfulness AW
*Word specificity*	Polysemy	Polysemy verbs
	Hypernymy	Hypernymy nouns and verbs

### 3.4. Data collection and analysis

The study was positioned in the Comprehensive English course which lasted for one academic year, and the participants were assigned a timed writing task every three or 4 weeks in class. They were instructed to work individually, and the use of a dictionary or any other reference material was prohibited. At the end of the first semester, the participants composed four compositions (two argumentative and two expository compositions). The remaining four compositions were collected in the second semester. Altogether, there were a total of 432 compositions for analysis.

To address the first research question, descriptive statistics were generated for lexical measures over time. Then, a one-way within-subjects ANOVA was conducted to capture the changes in lexical density, lexical variation, and lexical sophistication in argumentative and expository compositions, respectively. To address the second research question, a paired sample *t*-test was conducted to investigate whether differences existed concerning lexical richness in the two genres at the first time point of data collection. After that, a one-way repeated measures ANOVA was performed to examine the effects of genre on lexical richness across four time points over the academic year.

## 4. Results

### 4.1. Changes in lexical richness in argumentative and expository compositions over one academic year

In this section, the changes in lexical richness in argumentative and expository compositions are presented separately. First, the descriptive statistics of lexical richness in argumentative writing will be displayed along three dimensions: lexical density (LD), lexical variation (LV), and lexical sophistication (LS).

Table 3 shows that the mean values of LD in argumentative writing are significantly different across the four time points [*F*(3,162) = 41.067, *p* < 0.05]. There is a sharp increasing tendency from Time 1 to Time 2, then a slight decreasing tendency from Time 2 (0.508) to Time 3 (0.495), which is followed by another slight increasing tendency from Time 3 to Time 4 (0.501). The general changing tendency indicates that LD increases from the first writing to the fourth writing, though with a non-linear developmental pattern. Then, repeated contrast tests were conducted to assess how the four time points differed from one another, with the result of a statistically significant difference between Time 1 and Time 2 [*F*(3,162) = 32.558, *p* = 0.000].

**Table 3:** Changes in LD in argumentative writing over one academic year.

	Arg. 1	Arg. 2	Arg. 3	Arg. 4	*F*(3,162)
Lexical words	7,920	8,461	9,017	8,913	41.067*
All words	17,189	16,632	18,254	17,759	
Mean LD	0.461	0.508	0.495	0.501	
Std. Deviation	0.027	0.028	0.024	0.029	

Arg., argumentative; *p < 0.05.

The results indicate that lexical words account for more than half of the total words in L2 writers’ argumentative compositions, which in turn suggests that more information is produced in writing as students progress through the year, although the developmental pattern is non-linear. This finding is consistent with the developmental features of lexical density in L2 writing found by Bao (2008). One noteworthy point is that there was a slight decrease in LD at Time 3 compared with Time 2. The reason might be that the topic of the writing task influences the use of lexical words, as the topic of Time 2 was *Eating Habit*, while the topic of Time 3 was *Choice after Graduation*. Most sophomore students have not decided whether to find a job or pursue further studies. Therefore, they have a limited language repertoire about the topic and tend to use more functional words. The third argumentative writing assignment contained 1,066 more function words than the second one. The results show that the extensive use of function words decreases the value of lexical density.

The LV results exhibit a rising tendency in argumentative writing over one academic year. Table 4 indicates that the values of LV changed significantly over time [*F*(3,162) MATTR = 11.456; Maas = 13.019, *p* < 0.05]. The MATTR scores were positive for LV, while Maas scores calculated from the log correction approach were negative for LV. Though the changes in the two indices are upside down, they show the same developmental pattern in LV; that is, there is a small decrease in LV from Time 1 to Time 2, and a steady increase from Time 2 to Time 4. In order to determine whether the changes were significant, repeated contrast tests were conducted. The results indicate that a significant difference exists between Time 2 and Time 3 (*p* = 0.05) and between Time 3 and Time 4 (*p* = 0.001), while there is no significant difference between Time 1 and Time 2 (*p* = 0.34). This finding supports what has been concluded from previous studies: that is, as students gain knowledge in English, they perform better in L2 writing by using a great variety of words (Wang and Zhou, 2012; Zhu and Wang, 2013).

**Table 4:** Changes in LV in argumentative writing over one academic year.

Measure	Arg. 1	Arg. 2	Arg. 3	Arg. 4	*F*(3,162)
	M (SD)	M (SD)	M (SD)	M (SD)	
Tokens	17,189	16,632	18,254	17,759	
Types	8,479	8,212	9,097	9,184	
MATTR	0.784 (0.033)	0.782 (0.029)	0.790 (0.030)	0.806 (0.027)	11.456*
Maas	0.049 (0.006)	0.050 (0.006)	0.047 (0.006)	0.045 (0.006)	13.019*

Arg., argumentative; *p < 0.05.

Lexical sophistication (LS), as previously stated, is a multidimensional construct comprising frequency, word range, word property, and word specificity. Table 5 shows that the mean values of all the LS components were significantly different over one academic year. To be specific, the frequency of bigrams and academic words develops steadily with an overall upward tendency, and there are significant differences between adjacent times [*F*(3,162) AWL = 91.766; bigram = 11.306, *p* < 0.05]. The values of word range significantly decrease over time [*F*(3,162) KFR_CW = 101.879, *p* < 0.05], which indicates that the participants tended to use words occurring in fewer contexts. The developmental patterns of word familiarity and meaningfulness are quite similar. Repeated contrast tests reveal that there is a significant difference across the four time points [*F*(3,162), Familiarity = 21.021; Meaningfulness = 63.969, *p* < 0.05], which shows that EFL learners choose to use less familiar and less meaningful words in their writing as time goes on. Though the polysemy scores are significantly different across the four time points [*F*(3,162) Polysemy = 5.739, *p* < 0.05], the scores are almost the same at Time 4 compared with Time 1. Hypernymy scores tend to increase over time. As well, the scores are significantly higher at Time 2, and the scores decrease gradually at Time 3 and Time 4 [*F*(3,162) Hypernymy = 26.005, *p* < 0.05].

**Table 5:** Changes in LS in argumentative writing over one academic year.

Measure	Arg. 1	Arg. 2	Arg. 3	Arg. 4	*F*(3,162)
	Mean (SD)	Mean (SD)	Mean (SD)	Mean (SD)	
*Frequency*					
AWL	0.031 (0.015)	0.053 (0.024)	0.030 (0.013)	0.066 (0.019)	91.766*
Bigram	0.179 (0.042)	0.243 (0.091)	0.192 (0.068)	0.219 (0.076)	11.306*
*Range*					
KFR_CW	13.466 (0.361)	12.392 (0.494)	12.683 (0.454)	12.917 (0.439)	101.879*
*Word property*					
Familiarity	581.435	578.115	583.226	578.93	21.021*
	−3.997	−5.25	−5.08	−5.496	
Meaningfulness	356.861	351.245	362.537	344.576	63.969*
	−6.94	−11.51	−9.743	−8.378	
*Word specificity*					
Polysemy	11.029 (1.265)	10.707 (1.610)	10.154 (1.354)	11.102 (1.323)	5.739*
Hypernymy	3.917 (0.254)	4.274 (0.280)	4.201 (0.232)	4.183 (0.294)	26.005*

Arg., argumentative; *p < 0.05.

Table 5 shows an unstable increasing tendency in LS in argumentative writing. Nonetheless, a large number of previous studies have demonstrated an increase in LS over time (Bao, 2008; Wan, 2010; Wang and Zhou, 2012; Zhu and Wang, 2013; Bulté and Housen, 2014; Kalantari and Gholami, 2017). Compared with previous studies, the present study used many finer-grained indices of LS instead of choosing only the proportion of low-frequency words. For example, the indices of frequency in this study included bigram and word frequency, and the participants tended to more frequently use bigrams over one academic year. Examples 1 and 2 below highlight the differences between sentences with high and low bigram frequency scores.

1. High bigram frequency example (bigram score of 0.21): Perhaps some people’s views of dealing with difficulties rely on others’ suggestions and guidance.
2. Low bigram frequency example (bigram score of 0.13): Working earlier means experiencing worldly wisdom earlier than those who pursue further study.

The bigram frequency corpus adopted in TAALES contains the most frequent 50,000 bigrams of the BNC. Example 1 shows a comparatively higher score, for most of the bigrams in the sentence appear in the corpus. Of the 15 bigrams in Example 1, 12 can be found in the database. “Perhaps some suggestions and guidance” does not occur in the 50,000 most frequent bigrams in the BNC. In the second example, which gets a lower bigram score, only 4 of the 12 bigrams occur in the database. This evidence concurs with previous studies showing that advanced L2 learners tend to use more frequent bigrams (Salsbury, 2000; Crossley et al., 2010; Crossley and Skalicky, 2019) and lends support to the claim that bigram indices could more strongly indicate LS than word frequency in L2 writing (Kyle and Crossley, 2016).

Word range, word property, and word specificity are negative predictors of L2 writing proficiency (Kim et al., 2018). In the present study, they all (except for the hypernymy norm) showed a negative growth pattern, indicating that L2 learners began to produce words that were less familiar, less meaningful, less abstract, and occurred in a limited context. Moreover, the present study concurs with Kim et al.’s (2018) study, which explored lexical growth using LS components. As shown in Table 5, polysemy significantly increased over one academic year, which deserves special attention. High polysemy scores indicate that texts containing words with more senses are more ambiguous. Research has shown that as proficiency levels increase, L2 learners tend to produce words with fewer senses (Schmitt, 1998). Word specificity is closely related to frequency, and we explain it thoroughly in the section on genre differentiation.

Generally speaking, all indices significantly increased in argumentative compositions across the four time points of data collection. Similar findings have also been reported in Wang and Zhou’s (2012) study, where they explored the development of lexical richness of college students across three terms and yielded a stable increase in LD, LV, and LS. The results are also consistent with most studies that have reported advancement in the three constructs of lexical richness (Larsen-Freeman, 2006; Tan, 2006; Wan, 2010; Wang and Zhou, 2012). As with the changes in lexical richness in argumentative writing, the changes in expository writing will also be presented in terms of lexical density, lexical variation, and lexical sophistication.

For LD in expository writing, there is a weak rising tendency from Time 1 to Time 4, with some fluctuations in between. Table 6 shows that the scores of LD are significantly different across four time points [*F*(3,162) = 29.757, *p* < 0.05], and there is a slight decrease in LD from Time 2 to Time 3. Moreover, the results of repeated contrasts tests show that there are significant differences between adjacent times (*p* < 0.05), suggesting that the students tend to use more lexical words in their writing as their language proficiency improves.

**Table 6:** Changes in LD in expository writing over one academic year.

	Exp. 1	Exp. 2	Exp. 3	Exp. 4	*F*(3,162)
Lexical words	8,120	8,731	7,832	8,733	29.757*
All words	17,651	18,080	16,792	17,382	
Mean LD	0.46	0.484	0.467	0.503	
Std. Deviation	0.03	0.023	0.031	0.027	

Exp., Expository; *p < 0.05.

This finding aligns with Ravid (2004), who found that the LD of written expository texts increases over time. Nevertheless, there was a significant decrease in LD from Time 2 to Time 3. It might be that the third expository was written by the students at the beginning of the second semester after spending the winter vacation, and the students lacked enough language input.

For LV, there was no significant development from Time 1 to Time 4. Table 7 shows the developmental pattern of LV over 1 year, with the values of both MATTR and Maas being insignificantly different [*F*(3,162) MATTR = 0.370; Maas = 0.452, *p* > 0.05]. Moreover, the results of repeated contrasts tests show that there are no significant differences between adjacent times (*p* > 0.05).

**Table 7:** Changes in LV in expository writing over one academic year.

Measure	Exp. 1	Exp. 2	Exp. 3	Exp. 4	*F*(3,162)
	M (SD)	M (SD)	M (SD)	M (SD)	
Tokens	17,651	18,080	16,792	17,382	
Types	9,031	9,230	8,698	8,881	
MATTR	0.791 (0.032)	0.788 (0.028)	0.788 (0.036)	0.792 (0.031)	0.37
Maas	0.046 (0.005)	0.045 (0.005)	0.046 (0.006)	0.046 (0.006)	0.452

Exp., Expository; *p < 0.05.

This finding differs from what has been concluded in cross-sectional studies conducted by Ravid (2004) and Jeong (2017) which both examined the effects of narrative and expository genres on language development across three proficiency levels (novice, intermediate, and advanced). They found that lexical variation in expository texts increased with proficiency level. The reason might be that 1 year is not long enough to capture changes in LV.

For LS, the scores of all four sub-constructs are significantly different across time (*p* < 0.05) (Table 8). The frequency of bigram significantly increases over time [*F*(3,162) bigram = 2.993, *p* < 0.05]. Likewise, the frequency of academic words also increases over time [*F*(3,162) AWL = 66.356, *p* < 0.05], and it is significantly different between adjacent times. The developmental change in the word range exhibits a downward tendency [*F*(3,162) KFR_CW-12.015, *p* < 0.05], which indicates that the words the students use are in a limited range of context. The scores for word property were negative for LS. The scores of word familiarity and meaningfulness are significantly different between adjacent times [*F*(3,162) Familiarity = 29.330; Meaningfulness = 12.717, *p* < 0.05], which suggests that the students tend to use more familiar and more meaningful words in their compositions. Polysemy scores are negatively correlated to LS, the values of which significantly decrease over time [*F*(3,162) Polysemy = 11.668, *p* < 0.05]; while hypernymy scores are positively correlated to LS, the values of which significantly increase as time goes by [*F*(3,162) Hypernymy = 17.689, *p* < 0.05].

**Table 8:** Changes in LS in expository writing over one academic year.

Measure	Exp. 1	Exp. 2	Exp. 3	Exp. 4	*F*(3,162)
	Mean (SD)	Mean (SD)	Mean (SD)	Mean (SD)	
*Frequency*					
Bigram	0.178 (0.062)	0.230 (0.066)	0.197 (0.066)	0.224 (0.061)	2.993*
AWL	0.035 (0.014)	0.067 (0.021)	0.033 (0.014)	0.050 (0.020)	66.356*
*Range*					
KFR_CW	13.046 (0.439)	12.682 (0.457)	12.853 (0.460)	12.990 (0.451)	12.015*
*Word property*					
Familiarity	580.079	575.695	580.68	583.396	29.330*
	−5.533	−5.119	−5.104	−5.185	
Meaningfulness	352.816 (7.221)	349.781 (8.084)	358.387 (7.680)	353.059	12.717*
				−9.043	
*Word specificity*					
Polysemy	11.703 (1.654)	11.427 (1.473)	12.656 (1.804)	11.199 (1.331)	11.668*
Hypernymy	4.046 (0.223)	4.372 (0.262)	4.236 (0.274)	4.225 (0.262)	17.689*

Exp., Expository; *p < 0.05.

The findings of this study indicate that the students tended to use more bigrams, more academic words, words with fewer senses, less abstract and more specific words, but more familiar and meaningful words in expository writing. The four sub-constructs of LS in question develop in a balanced way, such as low polysemy but high familiarity and meaningfulness. This finding agrees with what was concluded by Zhu and Wang (2013) and Zhang et al. (2021); that is, lexical complexity is not an isolated phenomenon restricted to a stable domain; rather, whereas when LS increases, it causes a decrease in other dimensions of lexicon at different levels.

Overall, the picture of the developmental changes in lexical richness is not straightforward in Chinese EFL learners’ expository compositions. LD and LS showed a significant increase over the year, while no significant development was detected in terms of LV. The developmental tendencies are non-linear, which offers further support that the language complexity system is dynamic and complicated (Larsen-Freeman, 2006; Norris and Ortega, 2009).

### 4.2. Effects of genre on lexical richness in L2 writing

To probe into the effects exerted by genre on lexical richness, a paired sample *t*-test was conducted with genre as a within-subject variable. The values of the lexical richness measures for the two genres at four time points are displayed in Table 9.

**Table 9:** Lexical richness measures by time point and genre.

Measure	Arg.1 and Exp.1			Arg.2 and Exp.2			Arg.3 and Exp.3			Arg.4 and Exp.4		
	*MD*	*t*	*p*	*MD*	*t*	*p*	*MD*	*t*	*p*	*MD*	*t*	*p*
LD												
T_lex_/*T*	0.001	0.255	0.799	0.024	4.964	0.000*	0.029	6.305	0.000*	−0.002	−0.373	0.71
LV												
MATTR	−0.007	−1.53	0.132	−0.006	−1.271	0.209	0.003	0.532	0.597	0.015	2.797	0.007*
Maas	0.003	3.745	0.000*	0.004	4.813	0.000*	0.002	1.966	0.054	−0.001	−1.446	0.154
LS_Frequency												
AWL	−0.003	−1.474	0.146	−0.013	−3.558	0.001*	−0.003	−1.51	0.137	0.016	5.46	0.000*
Bigram	−0.047	−5.246	0.000*	0.013	0.971	0.336	−0.005	−0.434	0.666	−0.005	−0.403	0.689
Range												
KFR_CW	0.42	6.661	0.000*	−0.29	−3.825	0.000*	−0.17	−2.297	0.026*	−0.073	−1.181	0.243
Word property												
Familiarity	1.357	2.093	0.041*	2.42	2.663	0.010*	2.546	3.03	0.004*	−4.466	−5.922	0.000*
Meaningfulness	4.045	3.086	0.003*	1.464	0.828	0.412	4.15	2.916	0.005*	−8.482	−5.876	0.000*
Word specificity												
Polysemy	−0.675	−2.685	0.010*	−0.72	−2.573	0.013*	−2.502	−8.82	0.000*	−0.097	−0.406	0.686
Hypernymy	−0.129	−2.893	0.005*	−0.098	−1.826	0.073	−0.035	−0.817	0.417	−0.042	−0.904	0.37

*p < 0.05; Arg., Argumentative; Exp., Expository. Not significant with Bonferroni adjustment.

As demonstrated in Table 9, there was no significant difference in lexical density between genres at Time 1 [*t*(54) = 0.255, *p* = 0.799]. For LV, only *Maas* [*t*(54) = 3.745, *p* = 0.000] was significantly higher in expository compositions than in argumentative compositions. The *t*-tests for the different sub-dimensions of LS yielded significant differences in the bigram values [*t*(54) = −5.246, *p* = 0.000], KFR_CW [*t*(54) = 6.661, *p* = 0.000], Familiarity [*t*(54) = 2.093, *p* = 0.041], Meaningfulness [*t*(54) = 3.086, *p* = 0.003], and Hypernymy [*t*(54) = −2.893, *p* = 0.005], which indicate that LS is higher in expository compositions. Regarding the frequency level, there are more academic words in exposition, but the differences are not significant.

Unlike at Time 1, LD was significantly higher in argumentation than in exposition [*t*(54) = 4.964, *p* = 0.000] at Time 2. However, expository compositions contain more diversity of words than argumentative ones. For the varied subdimensions of LS, expository compositions contain more academic words and words with lower familiarity (AWL [*t*(54) = −3.558, *p* = 0.001], Familiarity [*t*(54) = 2.663, *p* = 0.010]), while argumentative compositions contain words occurring in fewer contexts and words with fewer senses {KFR_CW [*t*(54) = −3.825, *p* = 0.000]; Polysemy [*t*(54) = −2.573, *p* = 0.013]}. Other measures of LS showed no significant differences in argumentative and expository compositions.

The picture of Time 3 diverges from the first two time points. To be specific, LD, LV, and LS in both genres of writing decreased slightly when compared with Time 2. A possible reason might be that there was a long time span between the two writing times. There are significant differences in the measures of LD [*t*(54) = 6.305, *p* = 0.000], KFR_CW [*t*(54) = −2.297, *p* = 0.026], Familiarity [*t*(54) = 3.030, *p* = 0.004], Meaningfulness [*t*(54) = 2.916, *p* = 0.005], and Polysemy [*t*(54) = −8.820, *p* = 0.0003]. In contrast to the first three times of writing, the lexical richness was higher in argumentation than in exposition at Time 4 with regard to LV {MATTR [*t*(54) = 2.797, *p* = 0.007]}, Frequency {AWL [*t*(54) = 5.460, *p* = 0.000]}, and Word Property {Familiarity [*t*(54) = −5.922, *p* = 0.000]}; Meaningfulness [*t*(54) = −5.876, *p* = 0.000].

We also conducted a one-way repeated measures analysis of variance (ANOVA), with genre as an independent variable and the indices of lexical richness as dependent variables (see Table 10). The effect size was reported *via* partial eta squared (*ηp^2^*), with 0.0099 corresponding to a small effect size, 0.0588 corresponding to a medium effect size, and 0.1379 corresponding to a large effect size (Cohen, 1969). The ANOVA results indicate a significant genre effect on lexical density [LD *F*(1,432) = 23.813, *p* = 0.000, *ηp^2^ =* 0.052] and Polysemy [*F*(1,432) = 49.542, *p* = 0.000, *ηp^2^ =* 0.103] with medium effect sizes, suggesting that the students tend to use more lexical words and words with core senses in argumentative compositions than in expository ones. Regarding Word Property, there is no significant difference between the two genres. Nevertheless, a significant effect of genre was detected on LV and LS (except for Polysemy) with small effect sizes, namely Maas [*F*(1,432) = 10.665, *p* = 0.001, *ηp^2^ =* 0.024], Bigram [*F*(1,432) = 2.921, *p* = 0.048, *ηp^2^ =* 0.007], and Hypernymy [*F*(1,432) = 9.322, *p* = 0.002, *ηp^2^* = 0.021], indicating that expository compositions have more diverse words, more bigrams, and more specific words than argumentative ones.

**Table 10:** Genre effects on lexical richness measures.

Measure	Arg.	Exp.	*F*	*p*	*ηp^2^*
	Mean (SD)	Mean (SD)			
LD					
T_lex_/*T*	0.491 (0.033)	0.478 (0.033)	23.813	0.000*	0.052
LV					
MATTR	0.791 (0.031)	0.789 (0.032)	0.169	0.681	0
Maas	0.048 (0.006)	0.046 (0.006)	10.665	0.001*	0.024
LS_*Frequency*					
AWL	0.045 (0.024)	0.046 (0.022)	0.323	0.57	0.001
Bigram	0.208 (0.075)	0.219 (0.066)	2.921	0.048*	0.007
*Range*					
KFR_CW	12.864 (0.589)	12.893 (0.532)	0.44	0.508	0.001
*Word property*					
Familiarity	580.426(5.355)	579.962(5.893)	0.905	0.342	0.002
Meaningfulness	353.805(11.392)	353.510(8.562)	0.126	0.723	0
*Word specificity*					
Polysemy	10.748 (1.434)	11.746 (1.661)	49.542	0.000*	0.103
Hypernymy	4.144 (0.297)	4.220 (0.280)	9.322	0.002*	0.021

Arg., Argumentative; Exp., Expository; *p < 0.05.

## 5. Discussion

The results yielded from the present study bear on the two competing models reviewed in Literature Review, namely Robinson’s (2001) Cognition Hypothesis and Skehan’s (1998) Limited Attentional Capacity Model. According to the Cognition Hypothesis, an argumentative task is more complex than an expository task, so language is generally more complicated in argumentative writing than in expository writing. Some of our findings are consistent with those of previous studies, while others are not. The reason for this inconsistency might be that most previous studies adopted limited measures of lexical richness (especially lexical diversity) in comparing narrative compositions and non-narrative ones. The present study reveal that lexical density was higher for argumentative compositions than for expository ones. That is to say, the higher reasoning demand of the writing task pushes learners to use lexically denser language. This finding for lexical density partially supports Robinson’s Cognition Hypothesis. By contrast, according to Skehan’s (1998) Limited Attentional Capacity Model, argumentative writing involves a higher cognitive processing load compared to expository writing, so L2 learners would produce a language of higher lexical richness when composing expository compositions, which is cognitively less demanding. The results concluded in this study show greater lexical variation and lexical sophistication in expository compositions than in argumentative ones, which lends some support to the Limited Attentional Capacity Model.

Lexical variation, measured by MATTR and Maas in this study, is found to be significantly influenced by genre. Though MATTR shows no significant difference between the two genres, the value in argumentative compositions is still a little higher than in expository ones. By contrast, Maas shows a significant difference between argumentative and expository compositions. However, previous studies on lexical variation across genres (Yoon and Polio, 2017; Bi, 2020) found that learners would employ more diverse words in narrative essays than in argumentative ones. The lower lexical variation in argumentative compositions in this study might be attributed to two reasons. On the one hand, as the participants needed to revolve around the topic of the argumentative task, they would frequently use the words from the prompts to express their opinions. For example, the word “entertaining” and its derivation “entertainment” were used 186 times (3.4 times per composition), and in one composition it was used as many as 12 times. On the other hand, the participants showed greater reliance on the formulaic sequences that are common in argumentation. For instance, they were inclined to express personal views using formulaic phrases such as *in my opinion, from my perspective,* and similar expressions. In contrast, the expository writing prompts invited the writers to give reasons based on their personal experiences. By looking further into the participants’ compositions, we found that most of them chose to describe their personal experiences in detail. Higher lexical variation scores for expository compositions might be attributable to the use of vivid and engaging vocabulary, which is appropriate for personal experience.

The expository compositions exhibited greater lexical sophistication than the argumentative ones, which is beyond our expectation and contrary to the findings of most previous studies. According to Robinson’s (2007) Cognition Hypothesis, compared to expository writing, argumentative writing is more cognitively complex, and thus usually results in more complex language production. In analyzing the students’ compositions, we found that, in contrast to argumentative compositions, expository ones contained more advanced vocabulary (*sacrifice, compromise, reluctant, consensus, encounter, rebellion*) and longer words (*interpersonal, willingness, responsibilities, communication, discouragements*). In the present study, lexical sophistication is a multidimensional construct that includes frequency, range, word property, and word specificity. The use of advanced words and longer words discriminates the values of frequency and word property, for most of the unfamiliar and precise words are academic words occurring in limited contexts.

Many L2 studies have suggested a positive relationship between the percentage of less frequent words and L2 language proficiency (Daller et al., 2007; Kyle and Crossley, 2015). In view of the genre effects on frequency, most previous studies have found that argumentative compositions have more sophisticated or advanced words than narrative ones (Qin and Uccelli, 2016; Yoon and Polio, 2017). In this study, expository compositions contained more academic and bigrams than argumentative ones. Apart from frequency, word specificity, measured in terms of polysemy and hypernymy, also significantly distinguished argumentative and expository compositions. Moreover, as demonstrated in Table 10, the value of word range in argumentative compositions was lower than that in expository ones, which suggests that there were more sophisticated words in argumentative compositions, although the difference was insignificant. In addition, we can also find words of lower familiarity and meaningfulness in expository writing than in argumentative writing, despite insignificant statistical differences.

Finally, it should be pointed out that in L2 writing, there is no dichotomous classification for exposition and argumentation. Writing that involves personal judgment and opinions but does not take a side on something debatable is sometimes classified as exposition and, at other times, argumentation (Ravid, 2004). In the present study, exposition prompts were given to elicit personal opinions to explain or give reasons, so it is not surprising that learners used expressions conventional to argumentation, such as “from my perspective, I think, I believe, and in my opinion.” Some of the participants organized claims and arguments in a stepwise, hierarchical format, which is the typical way of organizing argumentative compositions. This might explain why the expository compositions contained more diverse (LV) and more sophisticated words (LS).

This study has some implications for L2 writing research methods, assessments, and instruction. Regarding the L2 writing research method, finer-grained indices should be taken into consideration in data collection. This study investigated a number of indices, such as range, familiarity, hypernymy, and polysemy, which all contribute to a deep understanding of lexical sophistication. To gauge the changes in lexical variation, this study used different measures to provide complementary, unique properties of the deployment of vocabulary in a text. Regarding L2 writing assessment, this study show that college-level EFL learners performed unequally well on the two writing genres, which points to the need to take into account the differential aspects of language pertaining to different genres when assessing students’ performance. Regarding L2 writing instruction, Chinese teachers often attach a great deal of importance to rote learning and memorization during foreign language education (Gong et al., 2021a). However, learning one set of discourse practices relevant to a particular context does not guarantee language performance in other contexts (Qin and Uccelli, 2016). Therefore, students should be taught to learn and acquire specific experiences, knowledge, and skills that are required in different professional and sociocultural roles (Gong et al., 2021b). Put another way, English teaching cannot be taken as just a way for promoting the overall language level and performance, which is, more often than not, believed to serve the same function in different contexts. Instruction based on specific genre contexts will provide a coherent framework for learning language and its use, thus ensuring that curriculum objectives come from students’ needs. Meanwhile, students should learn to develop their lexical richness along varied dimensions and at the same time enhance their awareness in relation to genre differences in L2 academic writing.

## 6. Conclusion

In response to the first research question, it was found that the subconstructs of lexical richness in argumentative and expository compositions developed in an unbalanced way over the course of one academic year. To be precise, argumentative compositions displayed a significant increase in lexical density, lexical variation, and lexical sophistication, while expository compositions exhibited a significant increase in lexical density and lexical sophistication, but not in lexical variation. Regarding the second research question, the results show that there were significant differences between argumentative compositions and expository ones on some of the measuring indices examined. To be exact, expository compositions display greater lexical variation and lexical sophistication than argumentative compositions do.

The findings yielded from the study lend support to both Robinson’s Cognition Hypothesis and Skehan’s Limited Attentional Capacity Model. On the one hand, the higher lexical density in argumentative writing than in expository writing partially supports Robinson’s Cognition Hypothesis (i.e., the higher reasoning demand of the writing task indeed pushes the learners to produce lexically denser language). On the other hand, the higher lexical variation and lexical sophistication in expository writing than in argumentative writing provide further evidence in support of Skehan’s Limited Attentional Capacity Model (i.e., L2 learners would produce language of higher lexical richness when composing expository writing, which is less cognitively demanding than argumentative writing).

As with most studies, the present study also has limitations. First, the study involved only a limited number of participants from the same class at a leading university. Thus, the results could by no means reveal the general developmental trajectories of lexical richness in writing. Second, comparing results from EFL learners with those of native speakers may yield more informative findings concerning the effects of genre on lexical richness. Third, the lexical richness measures investigated in this study failed to take accuracy into account, which makes a great difference in the quality of writing. Lastly, although one academic year is not short for a longitudinal study, it is still desirable to conduct similar research over a longer period of time.

## Data availability statement

The raw data supporting the conclusions of this article will be made available by the authors, without undue reservation.

## Ethics statement

The studies involving human participants were reviewed and approved by the School of Foreign Languages, Soochow University, Suzhou, China. The patients/participants provided their written informed consent to participate in this study.

## Author contributions

RH: conceptualization, research design, data analysis, and manuscript drafting. LP: research design, data analysis, drafting. XL data collection and data analysis. All authors contributed to the article and approved the submitted version.

## Conflict of interest

The authors declare that the research was conducted in the absence of any commercial or financial relationships that could be construed as a potential conflict of interest.

## Publisher’s note

All claims expressed in this article are solely those of the authors and do not necessarily represent those of their affiliated organizations, or those of the publisher, the editors and the reviewers. Any product that may be evaluated in this article, or claim that may be made by its manufacturer, is not guaranteed or endorsed by the publisher.
